# Prostaglandin Transporter (PGT/SLCO2A1) Protects the Lung from Bleomycin-Induced Fibrosis

**DOI:** 10.1371/journal.pone.0123895

**Published:** 2015-04-29

**Authors:** Takeo Nakanishi, Yoshitaka Hasegawa, Reo Mimura, Tomohiko Wakayama, Yuka Uetoko, Hisakazu Komori, Shin-ichi Akanuma, Ken-ichi Hosoya, Ikumi Tamai

**Affiliations:** 1 Faculty of Pharmaceutical Sciences, Institute of Medical, Pharmaceutical and Health Sciences, Kanazawa University, Kanazawa, Japan; 2 Faculty of Medicine, Institute of Medical, Pharmaceutical and Health Sciences, Kanazawa University, Kanazawa, Japan; 3 Department of Pharmaceutics, Graduate School of Medicine and Pharmaceutical Sciences, University of Toyama, Toyama, Japan

## Abstract

Prostaglandin (PG) E_2_ exhibits an anti-fibrotic effect in the lung in response to inflammatory reactions and is a high-affinity substrate of PG transporter (SLCO2A1). The present study aimed to evaluate the pathophysiological relevance of SLCO2A1 to bleomycin (BLM)-induced pulmonary fibrosis in mice. Immunohistochemical analysis indicated that Slco2a1 protein was expressed in airway and alveolar type I (ATI) and II (ATII) epithelial cells, and electron-microscopic immunohistochemistry further demonstrated cell surface expression of Slco2a1 in ATI cells in wild type (WT) C57BL/6 mice. PGE_2_ uptake activity was abrogated in ATI-like cells from *Slco2a1*-deficient (*Slco2a1* ^-/-^) mice, which was clearly observed in the cells from WT mice. Furthermore, the PGE_2_ concentrations in lung tissues were lower in *Slco2a1* ^-/-^ than in WT mice. The pathological relevance of SLCO2A1 was further studied in mouse BLM-induced pulmonary fibrosis models. BLM (1 mg/kg) or vehicle (phosphate buffered saline) was intratracheally injected into WT and *Slco2a1* ^-/-^ mice, and BLM-induced fibrosis was evaluated on day 14. BLM induced more severe fibrosis in *Slco2a1* ^-/-^ than in WT mice, as indicated by thickened interstitial connective tissue and enhanced collagen deposition. PGE_2_ levels were higher in bronchoalveolar lavage fluid, but lower in lung tissues of *Slco2a1* ^-/-^ mice. Transcriptional upregulation of TGF-β1 was associated with enhanced gene transcriptions of downstream targets including plasminogen activator inhitor-1. Furthermore, Western blot analysis demonstrated a significant activation of protein kinase C (PKC) δ along with a modest activation of Smad3 in lung from *Slco2a1* ^-/-^ mice, suggesting a role of PKCδ associated with TGF-β signaling in aggravated fibrosis in BLM-treated *Slco2a1* ^-/-^ mice. In conclusion, pulmonary PGE_2_ disposition is largely regulated by SLCO2A1, demonstrating that SLCO2A1 plays a critical role in protecting the lung from BLM-induced fibrosis.

## Introduction

Disordered eicosanoid synthesis has been reported in lung fibrosis in humans and rodents. Furthermore, increased leukotriene, but reduced prostaglandin (PG) E_2_ levels have been reported in bronchoalveolar lavage fluid (BALF) obtained from idiopathic pulmonary fibrosis (IPF) patients [1–3]. Since PGE_2_ in plasma is eliminated through pulmonary circulation [4], the lung is thought to be an important organ for metabolism of PGE_2_ that has escaped local inactivation. PGE_2_ can be synthesized in all types of cells in the lung [5–7], and has a well-documented role in homeostatic functions to protect alveolar epithelial cells from fibrotic injury. PGE_2_ decreases fibroblast proliferation and collagen production [8, 9], inhibits myoblast differentiation [10], and increases collagen degradation [11]. More severe fibrosis was observed in cyclooxygenase (*Cox)-2* ^-/-^ than in wild-type or *Cox-1* ^-/-^ mice when they were exposed to vanadium pentoxide [12] or bleomycin (BLM) [13]; however, other *in vivo* studies using *Cox-2* ^-/-^ [14, 15] and PGE receptor gene knockout mice [16] did not reproduce these findings.

PGE_2_ is synthesized through the COX/PGE synthase (PGES) pathway, and mediates diverse biological actions, including inflammatory responses. Extracellular PGE_2_ is taken up by cells and is then metabolized by cytoplasmic 15-hydroxyprostaglandin dehydrogenase (15-PGDH) [17, 18]. Rat hepatic Slco2a1, designated originally as organic anion transporting polypeptides (Oatp)2a1, has been characterized as PG transporter (PGT) with high affinity for PGE_2_ [19], and studies suggest that it plays a role in local disposition of PGs in mammals [20]. Previously, SLCO2A1 was suggested to function in vascular endothelium [21], gastroduodenal mucosa [22], choroid plexus [23] and retinal pigment epithelium [24]. Further, *SLCO2A1* gene expression is coordinately regulated with COX [25]. Therefore, SLCO2A1 may affect the actions of PGE_2_ in relation to tissue degeneration processes, such as fibrosis.

Relatively high mRNA expression of *SLCO2A1* was found in the lungs of humans [26] and mice [27], and SLCO2A1 protein is expressed in type II alveolar epithelial (ATII) cells [28]. Recently, we reported that SLCO2A1 is expressed in BEAS-2B human airway epithelial cells, where it may serve as a regulator of extracellular PGE_2_ at its site of action in response to inflammatory stimuli [29]. Nevertheless, expression of functional SLCO2A1 and the pathophysiological significance of SLCO2A1 in the lung are not fully understood. Therefore, the present study was designed to investigate the role of SLCO2A1 in PGE_2_ disposition by means of a study of BLM-induced pulmonary fibrosis in *Slco2a1* ^-/-^ and wild-type (WT) mice. Our results, including comprehensive analysis of Slco2a1 expression in the lung, indicate that SLCO2A1 is a major contributor to PGE_2_ uptake by type I alveolar epithelial (ATI) cells. Interestingly, we discovered that *Slco2a1* ^-/-^ mice exhibit more severe fibrosis, characterized by exacerbated collagen deposition and activation of protein kinase C (PKC) δ, as compared with WT, indicating that SLCO2A1 may be an independent determinant of tissue fibrosis. Therefore, the present study reveals a physiological significance of SLCO2A1, because it protects lungs from fibrosis in normal tissue homeostasis.

## Materials and Methods

### Materials and Animals

PGE_2_ and d_4_-PGE_2_ were purchased from Cayman Chemicals & Co. (Ann Arbor, MI). Dibutylhydroxytoluene was purchased from Wako Pure Chemical Industries (Osaka, Japan). An SLCO2A1 inhibitor, TGBz, was obtained from Ono Pharmaceutical Co., Ltd. Anti-mouse Slco2a1, Pges and 15-Pgdh IgGs were prepared as previously described [23, 30]. All other compounds and reagents were obtained from Sigma-Aldrich Company (St. Louis, MO), Life Technology (Carlsbad, CA), Wako Pure Chemical Industries, or Nacalai Tesque (Kyoto, Japan). Male Wistar rats and C57BL/6 mice were purchased from Sankyo Labo Service (Tokyo, Japan) and housed three and five animals per cage, respectively, with free access to commercial chow and tap water. They were maintained on a 12 h dark/light cycle (8:45 a.m.–8:45 p.m. light) in an air-controlled room (temperature, 23.0 ± 2°C; humidity, 55 ± 5%).

### Ethics Statement

All animal experimentation was carried out in accordance with the requirements of Kanazawa University Institutional Animal Care and Use Committee, and the protocols for animal experiments performed in this study were approved by the committee (Permit number, 72307, 73162, and 73163).

### *Slco2a1* ^-/-^ Mice

*Slco2a1* ^-/-^ mice were prepared and maintained as described [28]. Mice (C57BL/6), which carry a floxed allele of *Slco2a1* exon1 flanked with *LoxP* sites (S1 Fig), were generated and designated as *Slco2a1* ^flox/+^ mice. *Slco2a1* ^flox/flox^ mice were crossed with *Slco2a1* ^+/-^ mice, which carry Cre transgene under control of chicken beta actin promoter/enhancer coupled with the cytomegalovirus (CMV) immediate-early enhancer (B6;CBA-Tg(CAG-Cre)47lmeg, CAG-Cre), and then *Slco2a1* ^-/-^ offspring mice were obtained. Genotype and mRNA expression of *Slco2a1* were confirmed (S2 and S3 Figs).

### Immunohistochemistry

Immunohistochemical examination was basically carried out as described previously [31]. After acclimation, WT (C57BL/6) mice (23.4 ± 0.65 g, at age of 7 to 9 weeks) were anesthetized with an intraperitoneal (i.p.) injection of pentobarbital sodium (50 mg/kg), and sacrificed by exsanguination. Lung tissues were excised, and then fixed with 4% paraformaldehyde. Briefly, for light-microscopic analysis, frozen or paraffin-embedded sections were incubated with rabbit anti-Slco2a1 IgG (1:100, overnight at 4°C) [23], guinea pig anti-Slco2a1 IgG (1:20, overnight for 4°C) [23], guinea pig anti-Pges-1 IgG (1:5, 1 h at room temperature (rt)) [30], rabbit anti-15-Pgdh IgG (1:50, for 1 h, rt) (Cayman Chemical, Ann Arbor, MI), or rabbit anti-pro-surfactant protein C (SPC) serum (1:2000, 1 h at rt) (Millipore, Billerica, MA), and then successively reacted with biotinylated or fluorescence-labeled secondary antibody (1:200–400, 1 h at rt). For DAB staining, the biotinylated IgG labeled-sections were reacted with horseradish peroxidase-conjugated streptavidin, and developed with DAB (Vector Laboratories, Burlingame, CA).The anti-Slco2a1 antibody was preabsorbed with blocking peptide for 1 h at rt. Electron-microscopic assays were performed as described previously [32]. The DAB-stained sections were postfixed in 1% OsO_4_ for 30 min, reacted with 1% uranyl acetate for 20 min, dehydrated and embedded in Glicidether 100 (Selva Feinbiochemica, Heidelberg, Germany). The sections were observed with a Hitachi H-7650 electron microscope (Tokyo, Japan).

### Isolation of ATII Cells and PGE_2_ Uptake

Male Wistar rats (170–210 g body weight, at the age of 8 weeks), and WT (C57BL/6) and *Slco2a1* ^-/-^ mice (24.8 ± 0.54 g body weight at the age of 7 to 9 weeks) were i.p. injected with pentobarbital sodium (50 mg/kg), and given heparin (500 U) via the jugular vein. ATII cells were prepared as described by Ikehata et al [33, 34]. Three rats and one or two mice (for each line) were used for one preparation to obtain 3 × 10^6^ and 0.5 × 10^6^ cells, which were required for minimum experiments, respectively. Preparations were repeated three times, respectively, in the present study. In general, a cannula was made in the trachea after a tracheotomy was performed, and then the postcaval vein was cut. Subsequently, lungs were perfused with physiological saline through the right ventricle, excised and further lavaged several times to remove macrophages. The lungs were filled with the solution B containing trypsin (133 mM NaCl, 5.2 mM KCl, 1.89 mM CaCl_2_, 1.29 mM MgSO_4_, 2.59 mM phosphate buffer, 10.3 mM HEPES, 5.6 mM glucose, and 0.25% (w/v) trypsin). After removal of the trachea, bronchi and large airways, the lung tissues were minced into small pieces and then treated with DNase I (250 μg/mL) in solution A (133 mM NaCl, 5.2 mM KCl, 2.59 mM phosphate buffer, 10.3 mM HEPES, and 5.6 mM glucose). The resultant cell suspension was overlaid on heavy and low density of Percoll solution as described previously [33, 34], and then ATII cells were obtained by centrifugation at 250 × g and 4°C for 20 min. The cells were prepared from animals, and cultured for 2 and 6 days in Dulbecco’s modified Eagle’s medium supplemented with 10% fetal bovine serum [33, 34]. [^3^H]PGE_2_ uptake was measured as described previously [35].

### BALF Collection and Tissue Preparation

Male WT (C57BL/6) and *Slco2a1* ^-/-^ mice (four mice per each group, at the age of 7 to 9 weeks) were i.p. injected with pentobarbital sodium (50 mg/kg). Under the anaesthetization, BALF collection was performed with two to three 0.5-ml aliquots of physiological saline, and the rate of recovery was more than 80% for each animal. Lavaged lung tissues were perfused with PBS and then excised to prepare tissue homogenates, and frozen or paraffin-embedded sections.

### LC-MS/MS Analysis of PGs

PGE_2_ extracted from tissue homogenates or BALF was quantified with LC-MS/MS. Tissue samples were mechanically homogenized with a homogenizer (Ultra-Turrax T25, IKA Japan, Osaka) in the presence of dibutylhydroxytoluene (w/v 1%) and d_4_-PGE_2_ as an internal standard. Crude lipid was extracted from the homogenates or the recovered BALF with hexane, and then formic acid (v/v 2%) was added to each sample and PGs were extracted with chloroform. PGE_2_ in the resultant residue was reconstituted with mobile phase consisting of 0.1% formic acid/acetonitrile (1:1, v/v). PGE_2_ was separated with an LC-20AD ultra-fast liquid chromatography system (Shimadzu Co., Kyoto, Japan) equipped with an analytical column (Mercury MS, C18, 20 × 4.0 mm, Luna 3 μm, Phenomenex, Torrance, CA) and quantified by mass spectrometric analysis with an API 3200TM triple quadrupole mass spectrometer (AB Sciex, Foster City, CA). Gradient elution was performed using mobile phase composed of 0.1% formic acid (A) and acetonitrile (B) at a flow rate of 0.3 ml/min. The gradient profile was 25–99% B for 0–5 minutes, 99% B for 5–7 minutes and 95–25% B for 7–9 minutes. The analytes were detected using electrospray negative ionization with monitoring of the mass transitions m/z 351.1→271 for PGE_2_ and m/z 355.1→275.2 for d_4_-PGE_2_. Analyst software version 1.6 was used for data manipulation.

### BLM-induced Mouse Pulmonary Fibrosis Model

BLM or vehicle (PBS) was intratracheally injected into four WT (C57BL/6) and five *Slco2a1* ^-/-^ mice (male at the age of 11 to 12 weeks) anesthetized by i.p. injection of pentobarbital sodium (50 mg/kg), which were then kept for 14 days. During the experiments the animal weights were recorded every three days. In consideration of the unexpected toxicity of BLM to *Slco2a1* ^-/-^ mice, the dose of BLM was set at 1 mg/kg as the maximum dose without affecting survival is reported to be 2.2 mg/kg [36]. On day 14, the animals were anesthetized with pentobarbital sodium (50 mg/kg, i.p. injection), and BALF was collected. The animals were exsanguinated under anesthesia, and then lung tissues were excised for pathological examination and determinations of PGE_2_ levels. Pathological examination was performed by observation of hematoxylin and eosin-stained paraffin-embedded sections. Collagen was stained using Picrosirius Red Staining kit (Polysciences Inc., Warrington, PA). The area of stained regions was evaluated with ImageJ software [37].

### Real-time Quantitative RT-PCR (qRT-PCR) Analysis

RNA was extracted from the same animal sets used for BLM-induced pulmonary fibrosis model as described above. Total RNA was prepared with ISOGEN (Nippon Gene, Tokyo, Japan), converted to cDNA without being treated with DNase, and then subjected to qRT-PCR using Brilliant III Ultra Fast SYBR Green QPCR Master Mix (Agilent Technologies, Santa Clara, CA). Gene-specific sense and anti-sense primers used were 5’-ggacggtgcccattcagcca-3’ and 5’-aggttcactgtagccgtgtcca-3’ for *Slco2a1*, 5’-cttcgctggtgatgatgctc-3’ and 5’-gatgatgccgtgttctatcg-3’ for α-smooth muscle actin (Sma), 5’-tgtctatcaagggagtgtgtgc-3’ and 5’- caactggagtatttccgtgacc-3’ for basic fibroblast growth factor (*Fgf-2*), 5’-tatttggagcctggacacac-3’ and 5’-gtagtagacgatgggcagtgg-3’ for transforming growth factor (*Tgf)-β1*, 5’-gacgcatggccaagaagaca- 3’ and 5’-attgcacgtcatcgcacaca-3’ for *Col1a1*, 5’-atccggtaacaagggtgagc-3’ and 5’-acccattacaccagctctgc-3’ for *Cola1a2*, and 5’-tcctcatcctgcctaagttctc-3’ and 5’-actgtgccgctctcgtttac-3’ for plasminogen activator inhibitor (*Pai)-1*. mRNA expression of these genes was normalized to that of 18S rRNA, and then analyzed by 2^−ΔΔCT^ methods [38].

### Western Blot Analysis

After i.p. injection of pentobarbital sodium (50 mg/kg), six WT and *Slco2a1* ^-/-^ mice were exsanguinated, and lung tissues were excised to prepare total homogenates. An aliquot of the homogenates (20–50 μg) were separated by SDS polyacrylamide gel, and then electrotransferred onto a polyvinylidene difluoride membrane (Millipore) using the same method as described previously [29]. The blots were probed at 4°C overnight with the primary antibodies against Slco2a1 (rabbit or guinea pig IgG was used at final concentration of 0.1 μg/mL) [23], Cox2 (Cayman Chemical), 15-Pgdh (Cayman Chemical), glyceraldehyde-3-phosphate dehydrogenase, Smad3, phosho-Smad3 (S423/S425), AKT, phoshpo-AKT (S473), protein kinase C (PKC) α, phospho-PKCαβI/II (S638/S641), PKCδ and phospho-PKCδ/θ (S643/S676) (Cell Signaling Technology, Danvers, MA), respectively. Then, the blots were incubated with the appropriate secondary antibodies against rabbit or guinea pig IgG conjugated to horseradish peroxidase (Life Technologies). Corresponding expression was detected with electrochemical luminescence assay (Wako Pure Chemical Industries) and results of Western blot analysis were taken by the use of Light-Capture II (ATTO, Tokyo, Japan). Densitometric analysis of quantification for each band on the blots was performed using a CS analyzer (ATTO)

### Statistics

Data are given as the mean of values from at least three individual experiments with the standard error of the mean (SEM). Statistical analyses were performed with the unpaired Student’s t-test, and a probability of less than 0.05 (*p* < 0.05) was considered statistically significant.

## Results

**Fig 1:**
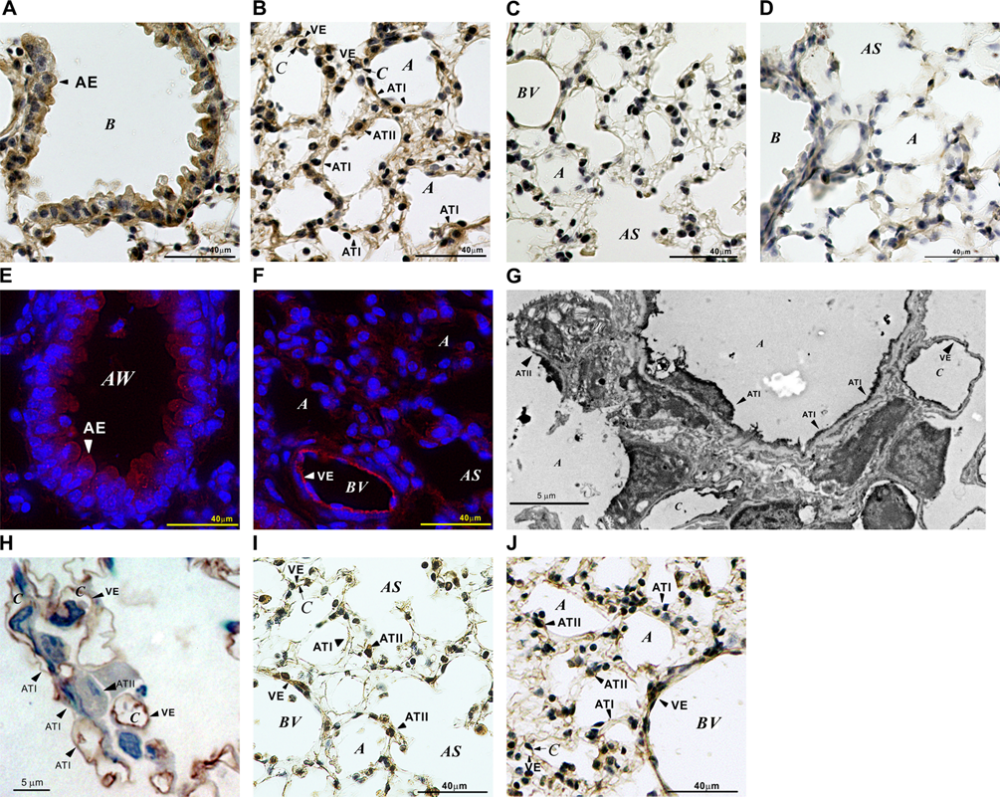
Immunohistochemical examination of Slco2a1 in mouse lung. (A-D) DAB immunohistochemistry was performed to examine Slco2a1 expression in mouse lungs. WT (A-C) and *Slco2a1* ^-/-^ (D) mouse lung cryosections (10 μm) incubated with anti-Slco2a1 antibody were stained brown by immunoenzymatic reaction with DAB in the absence (A, B, D) or presence of antigenic peptide (C). (E, F) Fluorescent immunostaining confirmed DAB staining of Slco2a1 expression. Sections were labeled with Alexa Fluor 594-conjugated secondary antibody and nuclei were stained blue with Hoechst 33342. (G) Electron-microscopic immunohistochemistry detected DAB staining of Slco2a1 in alveoli. (H) Semi-thin sections (4 μm) were also stained with DAB of Slco2a1. (I, J) DAB immunohistochemical analysis was performed to examine Pges (I) and 15-Pgdh (J) in the lung. Nuclei were counter-stained blue with hematoxylin (A-D, H-J). A, AE, AS, AW, B, BV, C and VE indicate alveoli, airway epithelial cells, alveolar sac, airway, bronchiole, blood vessel, capillary, and vascular endothelial cells, respectively.

Initially, Slco2a1 protein expression in lung tissue was examined by immunohistochemical approaches. Frozen mouse lung tissue sections were stained with anti-mouse Slco2a1 antibody and labeled with DAB. Immunoreactivity for the antibody was detected in the entire tissue, and intense DAB staining was observed at epithelium lining the respiratory tract (Fig 1A), vascular endothelial and alveolar epithelial cells (Fig 1B). Specificity of the immunoreactivity was confirmed by immune absorption of the primary antibody with synthetic mouse antigen peptides (Fig 1C), and the activity was diminished in lung sections prepared from *Slco2a1* ^-/-^ mice (Fig 1D). Fluorescent immunostaining gave similar results (Fig 1E and 1F). Slco2a1 protein seemed to be expressed in both ATI and ATII cells; therefore, its subcellular expression in alveoli was more closely studied by means of immunoelectron microscopy. DAB staining was primarily observed at the plasma membranes of ATI cells, whereas it was detected at cytoplasmic domain of ATII cells (Fig 1G). Cell surface expression of Slco2a1 was confirmed in ATI cell by DAB staining of semi-thin sections (Fig 1H). To understand SLCO2A1 function in relation to PGE_2_ synthesis and metabolism, expression of PGE_2_ synthase (Pges) and metabolic enzyme (15-Pgdh) was studied in the lung. Both enzymes were expressed in ATII and vascular endothelial cells (Fig 1I and 1J). Pges was primarily expressed in ATII, while 15-Pgdh expression was more strongly detected in vascular endothelial cells (Fig 1J).

**Fig 2:**
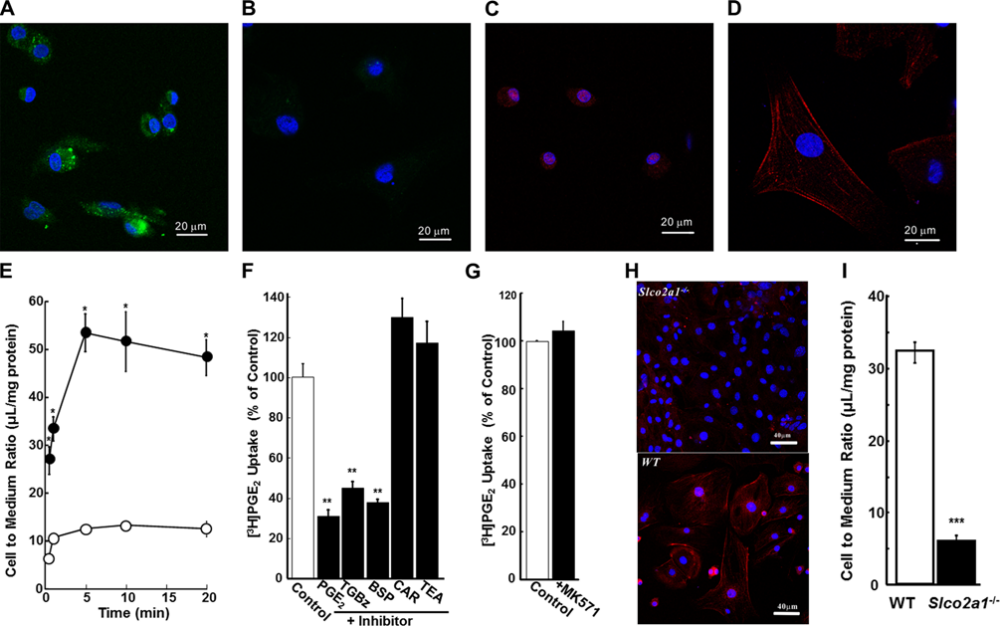
Expression of functional Slco2a1 in rat and mouse alveolar epithelial cells. (A-D) Fluorescent immunostaining for pro-SPC and Slco2a1 was performed in rat AECs in primary culture. Expression of pro-SPC (green) and Slco2a1 (red) was immunohistochmically detected in ATII (A, C) and ATI-L cells (B, D) primarily cultured from lung tissue of rats. (E) Uptake of [^3^H]PGE_2_ (3 nM) by ATII (open circles) and ATI-L (closed circles) cells in primary culture from rats was measured over 20 min at 37°C and pH 7.4 (F, G). The effect of various compounds on [^3^H]PGE_2_ (1.5 nM) uptake by rat ATI-L cells in primary culture was measured using unlabeled PGE_2_ (100 μM), TGBz (25 μM), BSP (a known inhibitor of SLCO2A1, 25 μM), CAR (carnitine, 1000 μM) and TEA (tetraethylammonium, 100 μM) for 5 min (F) and MK571 (25 μM) for 20 min (G). Uptake of [^3^H]PGE_2_ was normalized by the value obtained without any inhibitors (Control). (H) Immunofluorescence for anti-Slco2a1 (red) was confirmed in ATII and ATI-L cells (on Day 6) from *Slco2a1* ^-/-^ (top) and WT (bottom) mice. (I) [^3^H]PGE_2_ (3 nM) uptake by ATI-L cells from WT and *Slco2a1* ^-/-^ mice was measured. Each point or bar represents the mean ± SEM (at least n = 3). Student’s t-test was used for statistical analysis (*; p < 0.05, **; p <0.01, and ***; p<0.001).

Function of Slco2a1 was further examined in primary-cultured alveolar epithelial cells from rats and mice. Isolated round-shaped rat ATII cells differentiated into flat-shaped type-1 like alveolar (ATI-L) cells. ATII-characteristic expression of pro-SPC (Fig 2A) was lost in ATI-L cells on day 6 (Fig 2B). Immunofluorescence for Slco2a1 was detected mainly in the cytoplasm of ATII (Fig 2C), but at the plasma membranes of ATI-L cells (Fig 2D). The difference in subcellular localization of Slco2a1 expression between the two types of cell lines was reflected in [^3^H]PGE_2_ uptake activity, which was approximately 5 times higher in ATI-L cells than that in ATII cells (Fig 2E). The uptake was significantly decreased by inhibitors of OATP and SLCO2A1; however, it was not blocked by inhibitors of organic cation transporters or multidrug resistance associated protein, which is also known to transport PGE_2_ (Fig 2F and 2G). To further study the contribution of SLCO2A1 to the uptake by ATI cells, ATI-L cells were prepared from WT and *Slco2a1* ^*-/-*^ mice, respectively. Loss of cell surface expression of Slco2a1 was confirmed by lack of immunofluorescence in ATI-L cells (Fig 2H) and Western blot analysis using membrane fraction prepared from lung (S4 Fig), and [^3^H]PGE_2_ uptake was almost abrogated in these cells (Fig 2I), demonstrating its predominant contribution to absorption of PGE_2_ from alveolar lumen.

**Fig 3:**
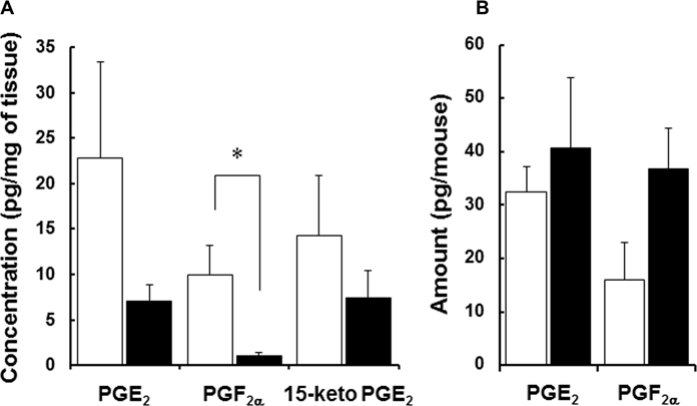
Disposition of PGE_2_ in the lung and BALF from WT and *Slco2a1*-/- mice. (A) Endogenous PGE_2_, PGF_2α_, and 15-keto PGE_2_ concentrations were analyzed using LC-MS/MS in lung homogenates of WT (open column) and *Slco2a1* ^-/-^ (closed column) mice. (B) Amounts of PGE_2_ and PGF_2α_ recovered in BALF were quantified by LC-MS/MS. Concentration was normalized by wet weight of tissues. Each column represents the mean (n = 4) + SEM. Student’s t-test was used for statistical analysis (*, p < 0.05).

To determine whether Slco2a1 affects pulmonary disposition of PGs, the amounts of PGE_2_ and another SLCO2A1 substrate PGF_2α_, as well as PGE_2_ metabolite 15-keto PGE_2_, were quantified in lung homogenates and BALF from WT and *Slco2a1* ^-/-^ mice. Tissue concentrations of PGE_2_ (p = 0.189) and 15-keto PGE_2_ (p = 0.36) tended to decrease and that of PGF_2a_ was significantly lower in *Slco2a1* ^-/-^, compared with WT mice (Fig 3A). Although no statistical changes were observed, the amounts of both PGE_2_ (p = 0.584) and PGF_2α_ (p = 0.114) in BALF were slightly increased in *Slco2a1* ^-/-^ mice (Fig 3B). Serum levels of PGE_2_ in WT and *Slco2a1* ^-/-^ mice were under the detection limit.

**Fig 4:**
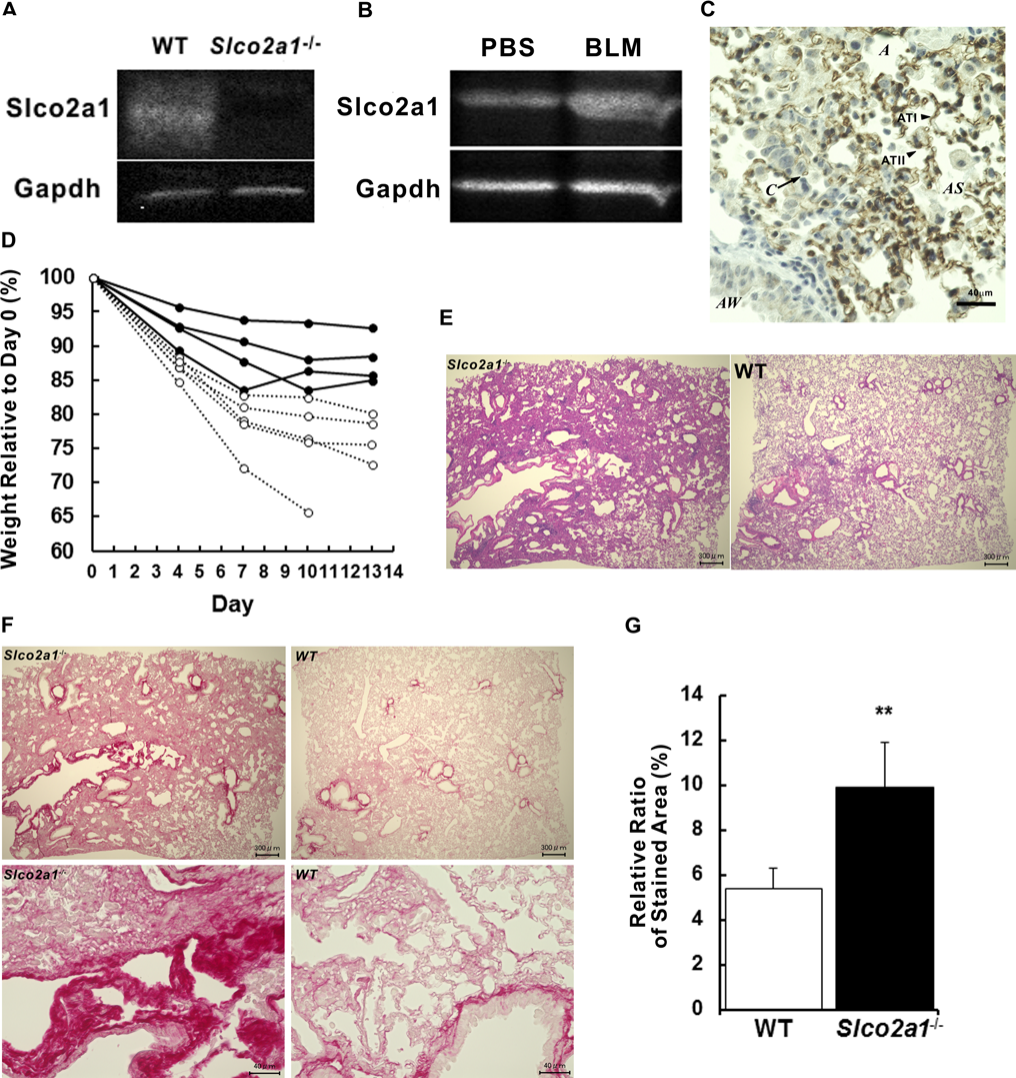
BLM-induced pulmonary fibrosis in WT and *Slco2a1*-/- mice. (A) Slco2a1 protein expression was confirmed by Western blot analysis in lung homogenates prepared from WT and *Slco2a1* ^-/-^ mice. (B) Slco2a1 protein expression was examined by Western blot analysis in lung homogenates from PBS- and BLM-treated WT mice. Western blot analysis was repeated at least three times using lung homogenates prepared from three mice, and a representative picture is shown. (C) Immunohistochemical analysis of Slco2a1 expression is shown in the lungs of BLM-treated WT mice. Figure shows a typical image of DAB staining with guinea pig anti-Slco2a1 antibodies. Nuclei were stained by hematoxylin. (D) Body weight of each WT (solid line, n = 4) or *Slco2a1* ^-/-^ (dotted line, n = 5) mouse is shown up to day 13. One *Slco2a1* ^-/-^ mouse died of severe fibrosis, and no other symptoms were observed. (E) Typical images of hematoxylin and eosin staining of lung sections are shown at low magnification (× 4); left panel shows *Slco2a1* ^-/-^ and right panel shows WT mice. (F) Typical images of Picrosirius Red staining of lung sections are presented at low (× 4, top panels) and high magnification (× 40, bottom panels). (G) The % of the area stained by Picrosirius Red was significantly increased in *Slco2a1* ^-/-^ (closed column), compared to the WT (open column) mice. Each bar represents the mean value of randomly selected 19 Picrosirius Red-stained images from at least 4 mice from each group. Student’s t-test was used for statistical analysis (**, p <0.01).

Since the lung concentration of anti-fibrotic PGE_2_ was likely reduced in *Slco2a1* ^-/-^ mice, we further studied the association of SLCO2A1 with pulmonary fibrosis induced by BLM. Western blot analysis confirmed that protein expression of Slco2a1 was absent in total lung homogenates from *Slco2a1* ^-/-^ mice (Fig 4A). In BLM-treated WT mice, total protein expression of Slco2a1 increased in lung (Fig 4B) and its protein expression was found to be mainly localized in alveolar epithelial cells rather than in stromal cells (Fig 4C). Intratracheal injection of BLM caused significant loss of body weight in both animal groups by day 3. By day 10, *Slco2a1* ^-/-^ mice including one dead animal had lost 24% of their initial body weight (28.16 ± 0.72 g), whereas WT had lost only 12.2% (the initial weight; 28.0 ± 1.57 g) (Fig 4D). Histological examination indicated more severe pulmonary fibrosis in *Slco2a1* ^-/-^ than in WT mice, with thickened interstitial connective tissue (Fig 4E). Enhanced collagen deposition in the tissues was observed by means of Picrosirius Red staining (Fig 4F), and the coverage ratio of the staining was significantly increased in *Slco2a1* ^-/-^ mice (Fig 4G).

**Fig 5:**
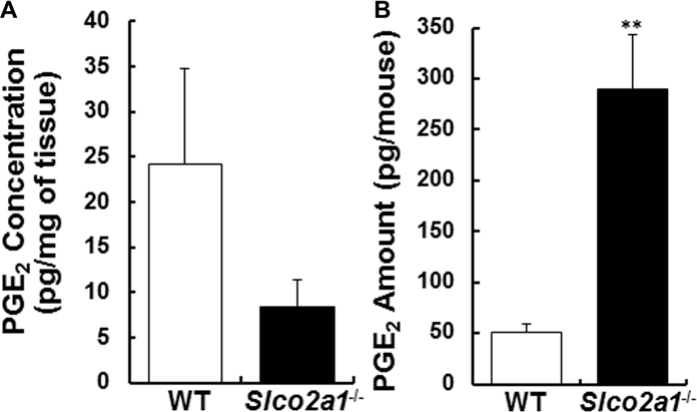
PGE_2_ levels in lung tissue homogenates and BALF of BLM-treated mice. (A) PGE_2_ concentrations were quantified in lung tissue homogenates of WT (open column) and *Slco2a1* ^-/-^ (closed column) mice. (B) Amounts of PGE_2_ in BALF were measured by means of LC-MS/MS as described in Material and Methods or S1 Table. Each column shows the mean of four individual determinants with SEM. Student’s t-test was used for statistical analysis (**, p<0.01).

To understand the relationship between PGE_2_ disposition and worsened fibrosis in *Slco2a1* ^-/-^ mice, the amounts of PGE_2_ in lung homogenates and BALF were quantified. PGE_2_ concentration in lung tissue homogenates tended to decrease (Fig 5A, p = 0.24), while the quantity of PGE_2_ recovered in BALF was greatly increased (Fig 5B, p = 0.008), compared to WT mice. However, there was no difference of PGE_2_ levels in lung homogenates and BALF between untreated and BLM-treated WT mice (Fig 3A and 3B). Metabolomics analysis showed that there were not any significant differences of other eicosanoids except for PGE_2_ in BALF between WT and *Slco2a1* ^-/-^ mice (S1 Table).

**Fig 6:**
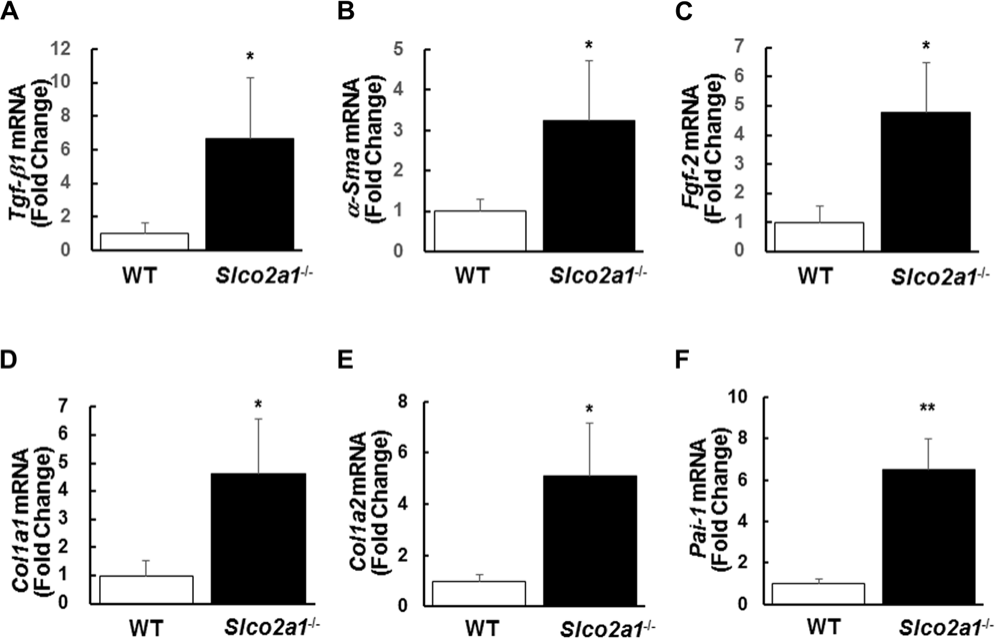
mRNA expression of fibrosis-related genes. Fibrosis-related gene expression was evaluated by quantifying mRNA expression; *Tgf-β1* (A), *α-Sma* (B), *Fgf-2* (C), *Col1a1* (D), *Col1a2* (E), and *Pai-1* (F). Each bar represents the mean of four individual determinants of WT (open column) or *Slco2a1* ^-/-^ (closed column) mice with SEM. Student’s t-test was used for statistical analysis (*, p < 0.05, **, p <0.01).

To characterize the aggravated fibrosis, we further analyzed gene expression of several fibrosis-related genes between BLM-treated WT and *Slco2a1* ^-/-^ mice. mRNA expression of transforming growth factor (*Tgf-β1*), and a typical marker for myofibroblast *α-Sma* was significantly increased in the lung of *Slco2a1* ^-/-^ mice treated with BLM (Fig 6A and 6B). In addition, *Fgf-2* was also transcriptionally upregulated (Fig 6C). Concomitantly, gene expression of major downstream targets of TGF-β signaling, *Col1a1*, *1a2* and *Pai-1* was enhanced, suggesting that TGF-β signaling is activated in the lung of *Slco2a1* ^-/-^ mice (Fig 6D–6F).

**Fig 7:**
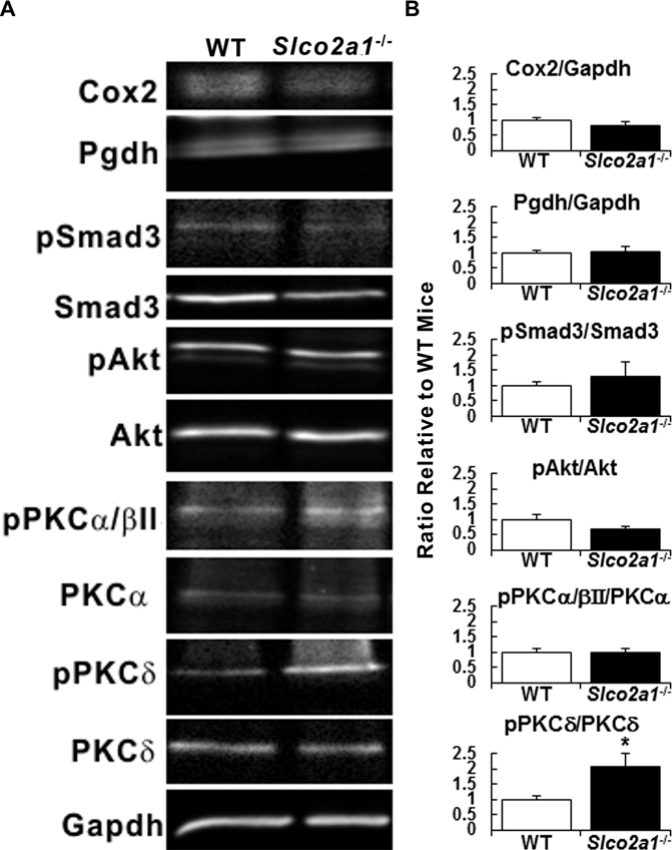
Western blot analyses of PGE_2_-related proteins and key signaling molecules in fibrosis. (A) Expression of proteins related to PGE_2_ metabolism and activation of signaling molecules were studied by Western blot analysis using lung homogenates prepared from WT and *Slco2a1* ^-/-^ mice (six per each group), and representative images are shown. (B) Quantitative analysis of each protein or phosphorylation was performed. Degree of expression of Cox2 and 15-Pgdh are shown by normalizing band intensity with that corresponding to Gapdh. Activation of phosphorylation of molecules was shown by normalizing band intensity for phosphorylated protein over the intensity for its total expression. Bands for phosphorylation of PKCδ and PKCθ were distinguished by molecular size on the blots. Phosphorylation of PKCα and βII was not distinguished by molecular size; therefore, we normalized their phosphorylation with total expression of PKCα and then the ratio was compared. Expression or degree of activation was compared between WT (open column) and *Slco2a1* ^-/-^ (closed column) mice, and each column represents the mean with SEM (lung tissues from 5 or 6 individual mice). Student’s t-test was used for statistical analysis (*, p < 0.05).

To look into a possible cause for the exacerbation of fibrosis observed in *Slco2a1* ^-/-^ mice, we finally studied expressions of PGE_2_-related proteins and activation of key signaling molecules between BLM-treated WT and *Slco2a1* ^-/-^ mice (Fig 7A). Statistical analyses of band intensities in Western blot analysis are shown in Fig 7B. Since protein expression of Cox-2 and 15-Pgdh was unchanged in the both lines of mice, only Slco2a1 could have contributed to pulmonary PGE_2_ disposition. Degrees of phosphorylation of Smad3, a key downstream regulator of TGB-β signaling, and Akt, which was important for proliferation of lung fibroblasts [39], were increased by 30.0% and decreased by 33.6%, respectively, in *Slco2a1* ^-/-^ mice, but their differences from those in WT mice did not reach the statistically significant level. PKCα, which was reported to mediate CC-chemokine ligand (CCL-18)-stimulated collagen production [40], was unlikely to be involved in the exacerbation of fibrosis in *Slco2a1* ^-/-^ mice because no change in phosphorylation levels was detected with antibody against PKCα/βII. Interestingly, phosphorylation of PKCδ, which has been implicated for collagen deposition in fibroblasts, was found to be elevated approximately two-times in *Slco2a1* ^-/-^, suggesting that activation of PKCδ is associated with the aggravated fibrosis observed in *Slco2a1* ^-/-^ mice.

## Discussion

In the present study, we found that SLCO2A1 was expressed in airway and alveolar epithelial cells in the lung, and functions as a transporter for PGE_2_ uptake by ATI cells. Slco2a1 deficiency resulted in retention of PGE_2_ in alveolar lumen and aggravated pulmonary fibrosis in mice treated with BLM. This is the first demonstration that impaired SLCO2A1 function is an independent determinant of tissue degeneration accompanied with fibrosis.

There is considerable evidence that defects of SLCO2A1 have pathogenic effects in humans. Finger or toenail clubbing often occurs in IPF patients, and recently missense mutations of *SLCO2A1*, including insertions of stop codons, have been found in patients with digital clubbing. Consequently, insufficient PGE_2_ clearance due to defective SLCO2A1 function is postulated to be the cause of hypertrophic osteoarthropathy [41], although the relationship between SLCO2A1 function and IPF remains unclear. Another study showed that myelofibrosis is involved in pachydermoperiostosis observed in *SLCO2A1*-deficient individuals [42], implying that failure of control of local PGE_2_ concentration is associated with tissue degeneration. These findings are consistent with our experimental observations regarding aggravation of pulmonary fibrosis.

First of all, we studied the pulmonary distribution of Slco2a1 protein in relation to PGE_2_ transport. In addition to ATII cells, where Slco2a1 was reported to be expressed in ATII cells in mice [28], it was found to be expressed in airway epithelial and ATI cells (Fig 1A–1H), especially at plasma membranes of ATI cells (Fig 1G and 1H). Interestingly, the cellular localization of Slco2a1 in ATI cells was distinct from that in ATII cells (Fig 2A–2D and 2H). Because PGE_2_ uptake by Slco2a1-deficient ATI-L cells was almost lost, it is thought that SLCO2A1contributes predominantly to pulmonary distribution of PGs by mediating PGE_2_ uptake by ATI cells, which cover 95% of the entire alveolar surface. As previously reported, Slco2a1 was also abundantly expressed in VE cells (Fig 1B and 1H) [21], where 15-Pgdh was highly detected (Fig 1J); therefore, SLCO2A1 may serve as uptake transporter for PGE_2_ from blood, which is important for systemic clearance of PGE_2_. In contrast to ATI cells, PGE_2_ uptake was not clearly observed in rat ATII cells (Fig 2E), where Pges was highly expressed (Fig 1I); therefore, SLCO2A1 might be involved in secretion or intracellular disposition, rather than cellular uptake, of PGE_2_ in the cells.

Freshly isolated ATII cells transdifferentiate to ATI-L cells and acquire ATI phenotype, which is defined by increased ability to transport small molecules and ions, in addition to flattened cell shape and ATI-specific gene expressions [43]. Water (aquaporin 5) and epithelial Na^+^ channel (ENaC) proteins are differentially upregulated in ATI cells as a part of the adoptive response to hypertonicity [44, 45]. Our study demonstrated for the first time that PGE_2_ uptake is mediated by Slco2A1 in ATI-L cells (Fig 2), and this result is consistent with characteristic change of cellular function of ATI cells. Loss of SLCO2A1 function caused an increase in PGE_2_ levels in BALF (Figs 3B and 5B), with levels of leukotrienes unchanged (S1 Table). Indeed, previous literature suggests that reactive oxygen species generated by BLM are involved in inflammatory reactions in the lung [46]. As the initial step, oxidation of arachidonic acid is triggered, and then active metabolites including PGE_2_ [47] and inflammatory cytokines are released, especially from alveolar macrophages [48, 49]. Taken together, our results suggest that increased PGE_2_ in alveolar lumen during BLM-induced lung injury might be efficiently absorbed to ATI cells via SLCO2A1 and that a part of the transported PGE_2_ is further translocated into interstitial tissue and then reutilized to compensate PGE_2_ shortage, which could have resulted from defective upregulation of COX-2 as reported in fibroblasts from IPF and rat BLM-induced fibrosis [3]. Namely, robust SLCO2A1 function in alveolar epithelial cells, especially ATI cells, may be essential for homeostasis of the lung and tissue remodeling. According to the hypothesized role of SLCO2A1, reduced levels of PGE_2_ reported in BALF of human IPF patients [1–3] might be explained by enhanced transport of PGE_2_ from alveolar lumen to interstitial tissues. As a part of pulmonary homeostasis, SLCO2A1 may contribute to protecting the lung from severe inflammation followed by fibrogenic reactions. This protecting role of SLCO2A1 could be supported by the following results: 1) total protein expression of Slco2a1 was increased in lung of BLM-treated WT mice (Fig 4B); 2) its localization was detected in alveolar epithelial cells (Fig 4C); and 3) *Slco2a1* ^-/-^ mice had severe weight loss when they were injected with BLM (Fig 4D)—the dose used was less than maximum and had no detrimental effect on survival [36]. Future work is warranted to determine alveolar lumen and interstitial tissues by the use of more sophisticated analytical techniques (e.g. microdialysis) during development of pulmonary fibrosis to prove this pathophysiological role of SLCO2A1.

In addition, decreased level of PGF_2α_ in lung (Fig 3A) may not contribute to the worse fibrosis because BLM-induced fibrosis was not aggravated in PGF receptor (FP) knockout mice [16]. However, PGF_2α_ was not detected in BALF in both BLM-treated WT and *Slco2a1* ^-/-^ mice (S1 Table). Furthermore, alteration of glutathione disposition in the lung might be involved in the exacerbation of fibrosis in *Slco2a1* ^-/-^ mice because such reducing agents ameliorate BLM-induced oxidation. Indeed, glutathione was suggested as a substrate of some OATP members [50]; however, our preliminary results indicated that glutathione is unlikely to be a substrate of SLCO2A1. Hence, involvement of glutathione may be ruled out.

In the present study, the two key genes for tissue fibrosis, *Tgf-β1* and *Fgf2*, were transcriptionally upregulated in the lung of *Slco2a1* ^-/-^ mice (Fig 6). FGF-2 is expressed in epithelial cells in the lung and released to extracellular matrix, where it contributes to proliferation and differentiation of fibroblast. Indeed, FGF-2 is secreted by alveolar epithelial cells in response to TGF-β1[51]. Besides, previous reports have suggested that PGE_2_ upregulates transcription of *FGF-2* [52], and promotes the mobilization of FGF-2 from membrane stores [53]. Since we observed increased levels of PGE_2_ in BALF in *Slco2a1* ^-/-^ mice, aberrant increase of PGE_2_ in alveolar lumen might result in over-expression or release of FGF-2 in ATI and/or ATII cells; thereby enhancing its action on fibroblasts.

Greater deposition of collagen fiber in lung of *Slco2a1* ^-/-^ mice may be explained by enhanced TGF-β signaling. TGF-β signaling plays a pivotal role in developing tissue fibrosis. In fibrotic response, classic signaling initiated by binding of TGF-β1 to TGF-β type II receptors is mediated by phosphorylation of Smad2 and Smad3, and then heterotrimeric complex formed with Smad4 enhances transcription of collagen and connective tissue growth factor by directly binding to their promoter regions [54]. The present study shows that gene expression of *Tgf-β1* and their downstream targets was upregulated (Fig 6); however, only moderate activation of Smad3 was detected in *Slco2a1* ^-/-^ mice (Fig 7). Accordingly, the enhanced gene expression of downstream targets of TGF-β signaling cannot be explained by the classic signaling pathway alone. Our results demonstrate that PKCδ was apparently activated in the lungs of *Slco2a1* ^-/-^ mice (Fig 7). Previous literature indicated that PKCδ is a possible participant in fibrotic TGF-β signaling in lung fibroblast [55]. Additionally, nonclassic pathway has been suggested independently, in which PKCδ activated by various kinases including c-Abl is associated with transcriptional upregulation of genes encoding collagen fibers and connective tissue growth factor, regardless of activation of Smads [56]. Considering a marginal activation of Smad3 (Fig 7), PKCδ is likely involved in greater collagen deposition in BLM-treated *Slco2a1* ^-/-^ mice, without depending upon activation of Smads. On the other hand, FGF-induced MAP kinase phosphorylation was reported to be mediated by PKCδ, but not PKCα [57]; therefore, activation of PKCδ may enhance action of FGF-2 in fibroblast. Because no significant changes were observed for activation of Akt and PKCα/βII, it is unlikely that they participate in aggravated fibrosis. At this moment, the mechanism underlying the activation of PKCδ in *Slco2a1*-dificient mice is under investigation. Since a previous report indicated that PKCδ activity was regulated in an EP3-dependent manner in primary endometriotic stromal cells [58], alteration of pericellular concentration of PGE_2_ by Slco2a1 deficiency may affect PKCδ activity in epithelial or stromal cells in the lung. Association of Slco2a1 function with PKCδ activity should be clarified in future based on accurate measurements of PGs including PGE_2_ and their metabolites because their changed or imbalanced disposition may be closely related to the state of fibrosis.

## Conclusions

SLCO2A1 is expressed in pulmonary epithelial cells, especially ATI cells, and is responsible for pulmonary disposition of PGE_2_. Since BLM-induced pulmonary fibrosis was exacerbated in *Slco2a1* ^-/-^ mice with Cox-2 and 15-Pgdh expression unchanged, SLCO2A1 itself is considered to be an independent determinant of local PGE_2_ concentration. Loss of function of SLCO2A1 could cause aggravation of pulmonary fibrosis, where activation of fibrotic signaling via PKCδ was involved in collagen deposition. These results suggest that SLCO2A1 functions to maintain PGE_2_ levels in alveolar lumen and interstitial tissues, indicating its critical role in lung tissue restoration processes during BLM-induced lung injury. Therefore, the present study demonstrates that SLCO2A1 protects the lungs from fibrosis and future studies are warranted to examine molecular mechanism underlying increased PKCδ signaling in *Slco2a1*-deficient mice.

## Supporting Information

S1 Fig *Slco2a1* exon 1-targeting knockout construct. For conditional *Slco2a1* knockout, *Slco2a1*-targeting knockout construct was designed according to the previous report [28]. Mouse genotyping using PCR showed that offspring carrying *Cre* transgene have knockout allele but lack floxed allele; thereby Cre/lox system successfully deletes exon 1 of *Slco2a1* gene located on mouse chromosome 9.
(PDF)

S2 Fig Genotyping of offspring. *Slco2a1* ^flox/flox^ mice were crossed with *Slco2a1* ^+/-^ mice, which carry *Cre* transgene under control of chicken beta actin promoter/enhancer coupled with the cytomegalovirus (CMV) immediate-early enhancer (B6;CBA-Tg(CAG-Cre)47lmeg, CAG-Cre), and then offspring mice were obtained. Genome DNA was prepared from tail of the offspring, and their genotypes were confirmed by polymerase chain reaction (PCR) using the designated sense primer for wild (primer A; 5’- AGGCTCTCGTGGGGAGTAAT -3’), floxed (primer B; 5’- AGTAGAAGGTGGCGCGAAG -3’) and knockout (primer C; 5’- AGGACCTGATAGGCAGCCAA -3’) alleles, respectively, with the same anti-sense primer D (5’- CACAGCAGAGACCCAACAGA -3’). Their locations were indicated in S1 Fig. Oligonucleotides specific to the *Cre* transgene were used for sense- (5’- ttacggcgctaaggatgact- 3’) and anti-sense (5’-ttgcccctgtttcactatcc-3’) primers to detect positivity of *Cre* gene. In general, PCR was performed in a 30 cycle of heat denature at 94°C for 15 sec, annealing at 58°C for 15 sec, and extension at 72°C for 30 sec, and amplified DNA fragments were analyzed by electrophoresis on 2% agarose gel and visualized with ethidium bromide. PCR analysis confirmed the four different genotypes in littermates. Mice that have neither wild nor floxed alleles of *Slco2a1* were defined as *Slco2a1* ^-/-^ mice.
(TIF)

S3 Fig mRNA expression of *Slco2a1*. mRNA expression was also studied in various tissues using gene specific primers for mouse *Slco2a1* exon1; sense, 5’-ccgctcggtcttcaacaac-3’ and anti-sense, 5’-aagaactggagagcccaaagc-3’, and amplified DNA fragments were compared with those in WT mice. Although expression of *Slco2a1* mRNA was confirmed in all tissues obtained from WT mice (lung, kidney, liver, colon, brain, testis and skeletal muscle); however, no expression was detected in *Slco2a1* ^-/-^ mice in all the tissues tested.
(TIF)

S4 Fig Protein expression of Slco2a1 in plasma membranes of the lung tissues. Crude membrane fraction from total lung tissue homogenates were prepared as described previously [30]. Western blot analysis was performed as described in Material and Methods. A single robust and thick band was found in WT, but not in that from *Slco2a1* ^-/-^ mice, demonstrating that Slco2a1 was at least expressed in the plasma membranes and the expression was abrogated in *Slco2a1* ^-/-^ mice.
(TIF)

S1 Table Metabolomic analysis of 48 eicosanoids in BALF in BLM-treated mice. Forty-eight lipid mediators and d_4_-PGE_2_ (internal standard) were mixed and diluted with ethanol:ultrapure water (1:1, v/v) to make 100 ng/mL standard solutions. BALF samples were diluted with 0.7 mL saline and adjusted to ethanol solution:BALF:formic acid (10:100:1, v/v/v) containing 4 ng internal d_4_-PGE_2_. Samples were transferred to solid-phase extraction cartridges (Empore 4 mm/1 mL C18 Standard Density, 3M). C18 cartridges were washed with 0.5 mL ethanol:ultrapure water:formic acid (10:100:1, v/v/v) and centrifuged at 5,000 rpm for 1 min at 4°C to remove water solution. Lipid mediators were eluted with 200 μL ethanol under centrifugation at 5,000 rpm for 1 min at 4°C. The solvent was evaporated in a centrifugal evaporator, and the residue was dissolved in 20 μL ethanol and diluted with 20 μL ultrapure water. Lipid mediators were measured with an Ultimate 3000 HPLC system (Thermo Fisher Scientific) combined with an API3200 QTRAP mass spectrometer (ABSCIEX). HPLC was conducted at 40°C using a *L-column2 ODS* (2.1 × 150 mm, pore size 2 μm, CERI). Samples were eluted with a mobile phase that comprised 5 mmol/L ammonium formate:formic acid (100:0.1, v/v) and acetonitrile in a 90:10 ratio for 1 min, followed by a ramp up to a 15:85 ratio after 26 min at a flow rate of 0.4 mL/min. Samples were kept at 5°C and 5 μL volumes were injected. MS-MS analyses were conducted in the electrospray ionization negative ion mode, and fatty acid metabolites were detected and quantified by multiple reaction monitoring. Source temperature was set for 400°C. The peaks were selected and their areas were calculated using Analyst 1.6.1 (ABSCIEX). Limit of detection was set at a signal/noise ratio of 3. Metabolomic analysis was performed in BALF collected from mice. Among 48 eicosanoids analyzed by means of LC-MS/MS method as described below, only PGE_2_, leukotriene D4 (LTD4), leukotriene E4 (LTE4), 14,15- DHET, 11,12-DHET, 11- hydroxyeicosatetraenoic acid (HETE), 15-OxoETE and 12-HETE were detected at significant levels. In addition to PGE_2_, 11-HETE tended to be increased in BALF from *Slco2a1* ^-/-^ mice.
(PDF)
