# Supplementary figures and images for: Prostaglandin Transporter (PGT/SLCO2A1) Protects the Lung from Bleomycin-Induced Fibrosis
Source: PLoS One. 2015 Apr 29;10(4):e0123895. doi: 10.1371/journal.pone.0123895 (PMC4414486; doi:10.1371/journal.pone.0123895)

S1\_Fig

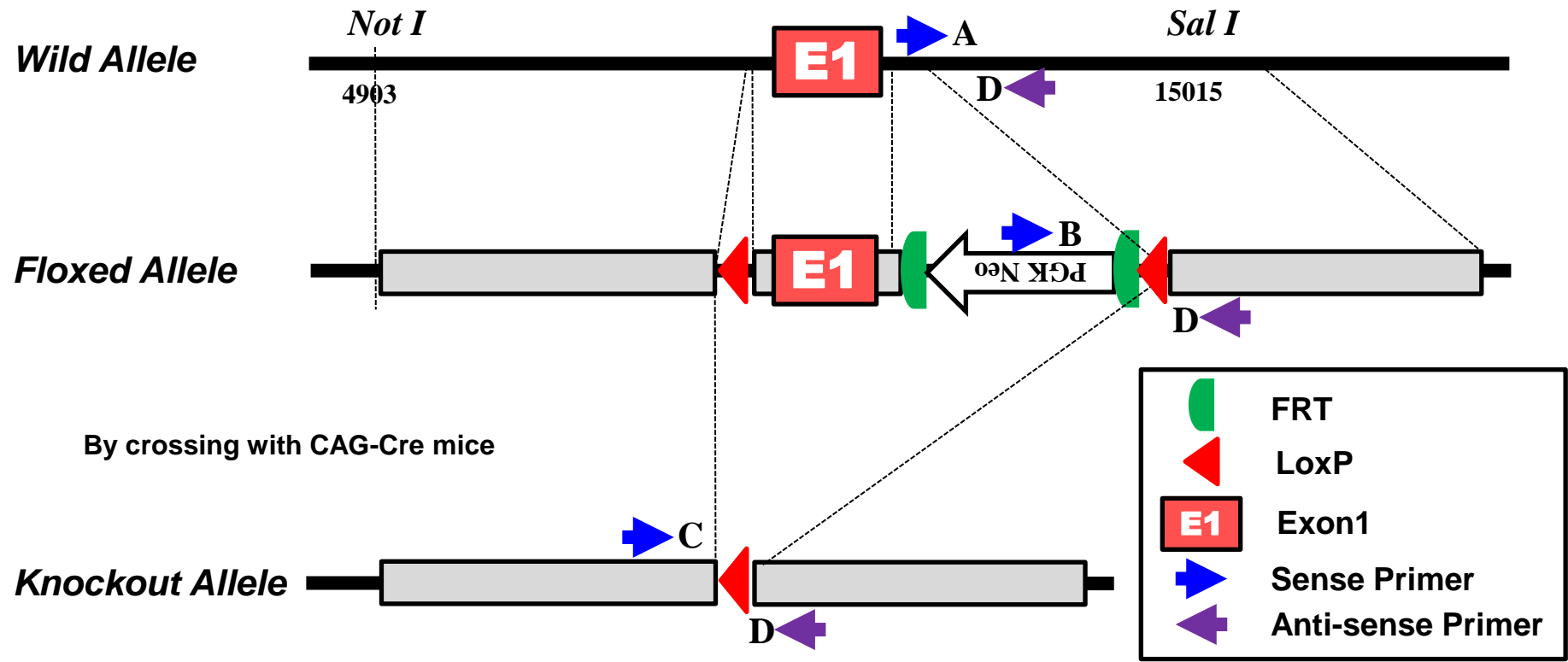

Supplement: S1 Fig — For conditional Slco2a1 knockout, Slco2a1-targeting knockout construct was designed according to the previous report [28]. Mouse genotyping using PCR showed that offspring carrying Cre transgene have knockout allele but lack floxed allele; thereby Cre/lox system successfully deletes exon 1 of Slco2a1 gene located on mouse chromosome 9. (PDF) [file pone.0123895.s001.pdf]

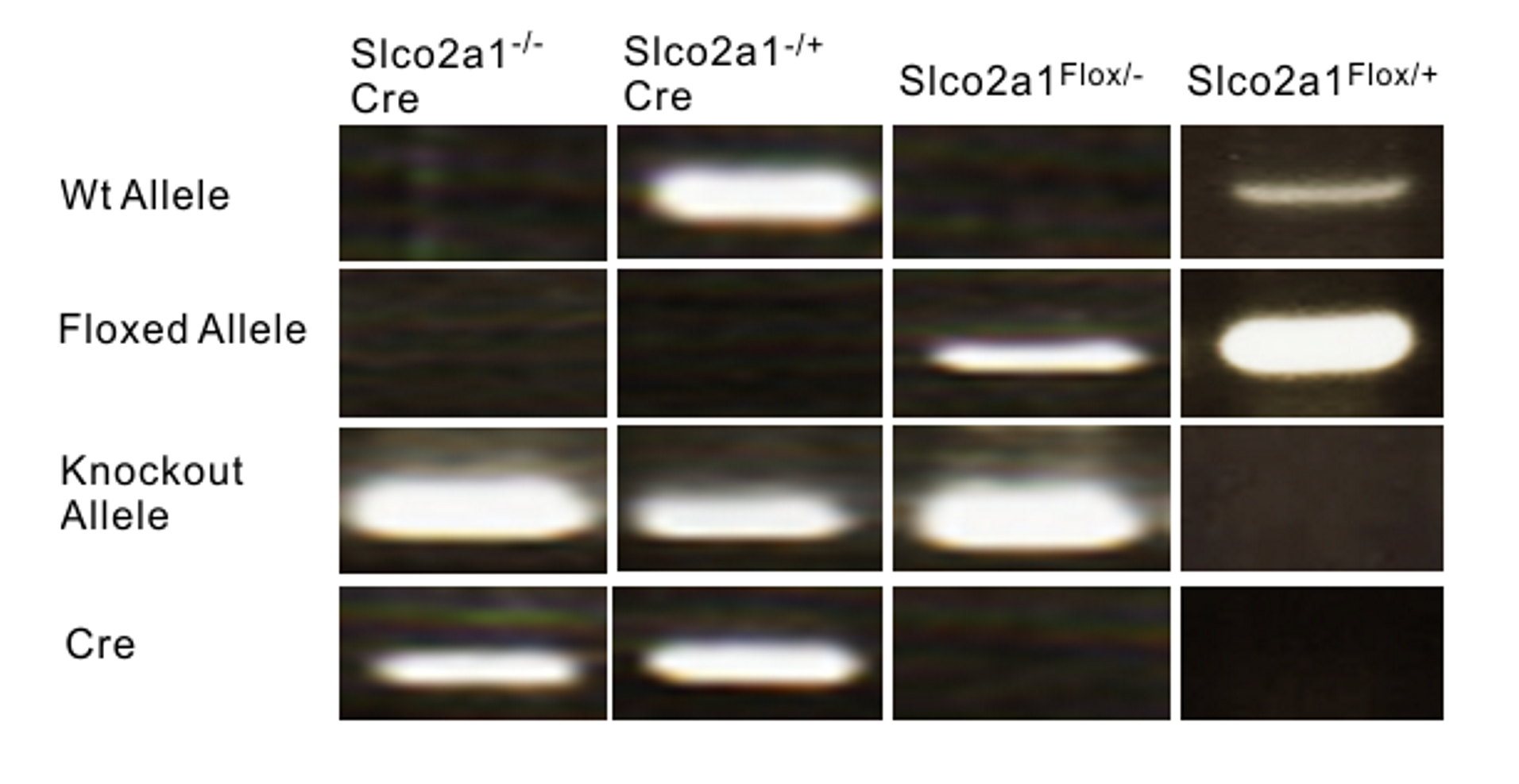

Supplement: S2 Fig — Slco2a1 flox/flox mice were crossed with Slco2a1 +/- mice, which carry Cre transgene under control of chicken beta actin promoter/enhancer coupled with the cytomegalovirus (CMV) immediate-early enhancer (B6;CBA-Tg(CAG-Cre)47lmeg, CAG-Cre), and then offspring mice were obtained. Genome DNA was prepared from tail of the offspring, and their genotypes were confirmed by polymerase chain reaction (PCR) using the designated sense primer for wild (primer A; 5’- AGGCTCTCGTGGGGAGTAAT -3’), floxed (primer B; 5’- AGTAGAAGGTGGCGCGAAG -3’) and knockout (primer C; 5’- AGGACCTGATAGGCAGCCAA -3’) alleles, respectively, with the same anti-sense primer D (5’- CACAGCAGAGACCCAACAGA -3’). Their locations were indicated in S1 Fig. Oligonucleotides specific to the Cre transgene were used for sense- (5’- ttacggcgctaaggatgact- 3’) and anti-sense (5’-ttgcccctgtttcactatcc-3’) primers to detect positivity of Cre gene. In general, PCR was performed in a 30 cycle of heat denature at 94°C for 15 sec, annealing at 58°C for 15 sec, and extension at 72°C for 30 sec, and amplified DNA fragments were analyzed by electrophoresis on 2% agarose gel and visualized with ethidium bromide. PCR analysis confirmed the four different genotypes in littermates. Mice that have neither wild nor floxed alleles of Slco2a1 were defined as Slco2a1 -/- mice. (TIF) [file pone.0123895.s002.tif]

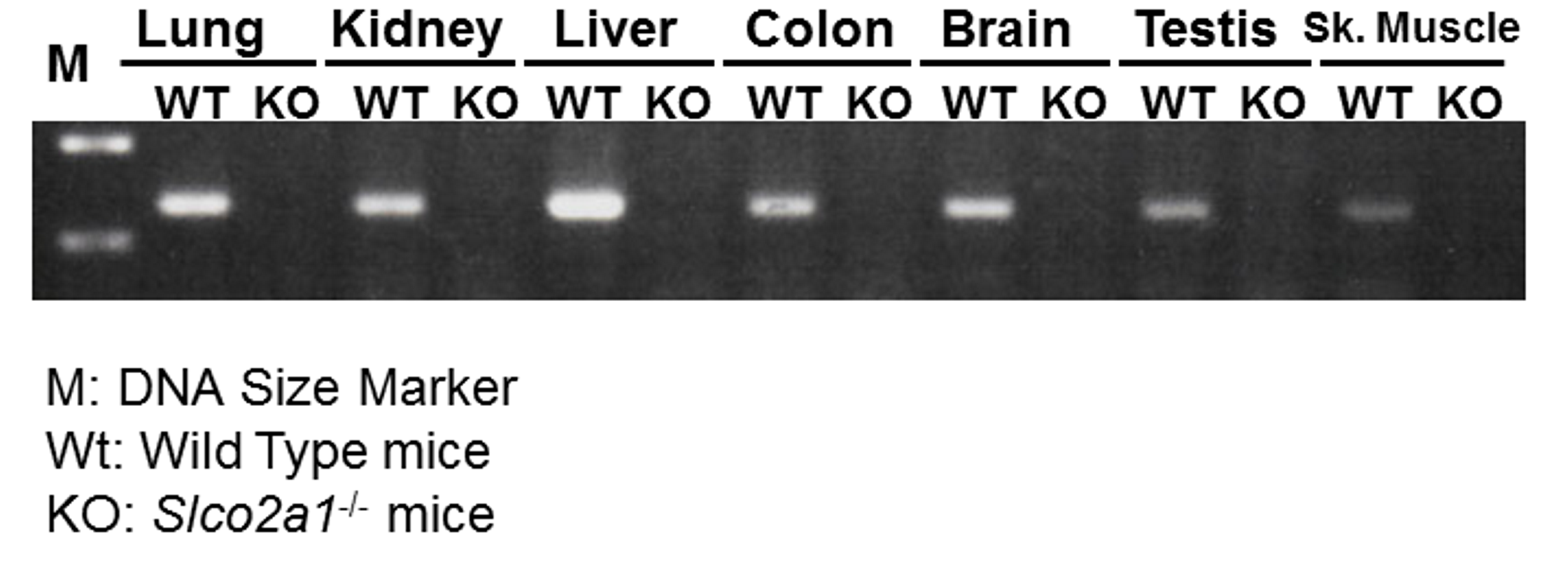

Supplement: S3 Fig — mRNA expression was also studied in various tissues using gene specific primers for mouse Slco2a1 exon1; sense, 5’-ccgctcggtcttcaacaac-3’ and anti-sense, 5’-aagaactggagagcccaaagc-3’, and amplified DNA fragments were compared with those in WT mice. Although expression of Slco2a1 mRNA was confirmed in all tissues obtained from WT mice (lung, kidney, liver, colon, brain, testis and skeletal muscle); however, no expression was detected in Slco2a1 -/- mice in all the tissues tested. (TIF) [file pone.0123895.s003.tif]

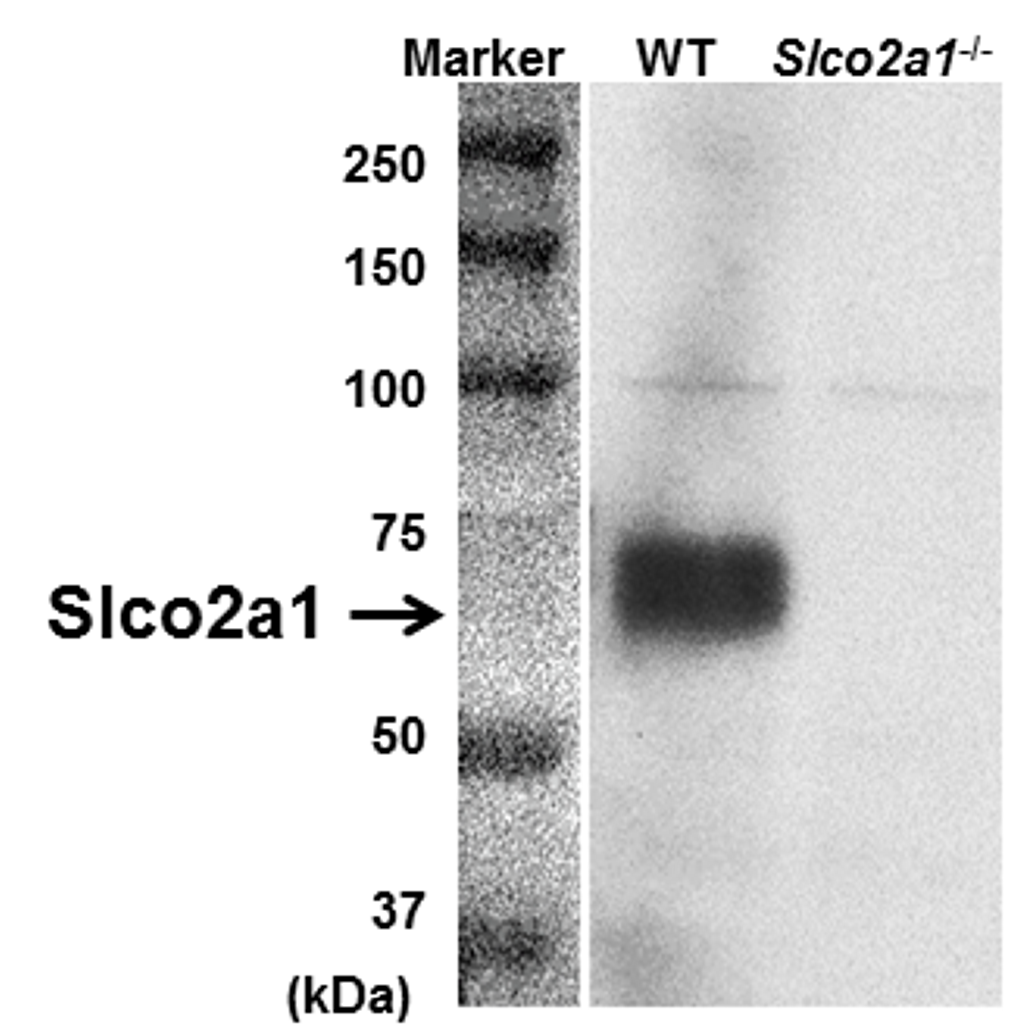

Supplement: S4 Fig — Crude membrane fraction from total lung tissue homogenates were prepared as described previously [30]. Western blot analysis was performed as described in Material and Methods. A single robust and thick band was found in WT, but not in that from Slco2a1 -/- mice, demonstrating that Slco2a1 was at least expressed in the plasma membranes and the expression was abrogated in Slco2a1 -/- mice. (TIF) [file pone.0123895.s004.tif]
