# Supplementary material for: Prostaglandin Transporter (PGT/SLCO2A1) Protects the Lung from Bleomycin-Induced Fibrosis
Source: PLoS One. 2015 Apr 29;10(4):e0123895. doi: 10.1371/journal.pone.0123895 (PMC4414486; doi:10.1371/journal.pone.0123895)
Supplement: S1 Table — Forty-eight lipid mediators and d4-PGE2 (internal standard) were mixed and diluted with ethanol:ultrapure water (1:1, v/v) to make 100 ng/mL standard solutions. BALF samples were diluted with 0.7 mL saline and adjusted to ethanol solution:BALF:formic acid (10:100:1, v/v/v) containing 4 ng internal d4-PGE2. Samples were transferred to solid-phase extraction cartridges (Empore 4 mm/1 mL C18 Standard Density, 3M). C18 cartridges were washed with 0.5 mL ethanol:ultrapure water:formic acid (10:100:1, v/v/v) and centrifuged at 5,000 rpm for 1 min at 4°C to remove water solution. Lipid mediators were eluted with 200 μL ethanol under centrifugation at 5,000 rpm for 1 min at 4°C. The solvent was evaporated in a centrifugal evaporator, and the residue was dissolved in 20 μL ethanol and diluted with 20 μL ultrapure water. Lipid mediators were measured with an Ultimate 3000 HPLC system (Thermo Fisher Scientific) combined with an API3200 QTRAP mass spectrometer (ABSCIEX). HPLC was conducted at 40°C using a L-column2 ODS (2.1 × 150 mm, pore size 2 μm, CERI). Samples were eluted with a mobile phase that comprised 5 mmol/L ammonium formate:formic acid (100:0.1, v/v) and acetonitrile in a 90:10 ratio for 1 min, followed by a ramp up to a 15:85 ratio after 26 min at a flow rate of 0.4 mL/min. Samples were kept at 5°C and 5 μL volumes were injected. MS-MS analyses were conducted in the electrospray ionization negative ion mode, and fatty acid metabolites were detected and quantified by multiple reaction monitoring. Source temperature was set for 400°C. The peaks were selected and their areas were calculated using Analyst 1.6.1 (ABSCIEX). Limit of detection was set at a signal/noise ratio of 3. Metabolomic analysis was performed in BALF collected from mice. Among 48 eicosanoids analyzed by means of LC-MS/MS method as described below, only PGE2, leukotriene D4 (LTD4), leukotriene E4 (LTE4), 14,15- DHET, 11,12-DHET, 11- hydroxyeicosatetraenoic acid (HETE), 15-OxoETE and 12-HETE were dete [file pone.0123895.s005.pdf]

S1Table.

No.	Compound	Amount in BALF (ng/mouse)				
		no.1 (WT)	no.2 (WT)	no.3 (WT)	no.4 (*Slco2a1*$^{-/-}$)	no.5 (*Slco2a1*$^{-/-}$)
1	2,3-Dinor-8-iso-PGF2α					
2	6-Keto-PGF1α					
3	20-COOH-LTB4					
4	6-Keto-PGE1					
5	20-OH-LTB4					
6	TXB2					
7	PGF2α					
8	PGE2	0.081	0.052	0.034	0.341	0.364
9	11-Dehydro TXB2					
10	15-Keto-PGF2α					
11	LXB4					
12	PGD2					
13	LXA4					
14	LTD4	0.009	0.003	0.003	0.005	0.008
15	LTC4					
16	LTF4					
17	LTE4	0.052		0.074	0.085	0.242
18	PGA2					
19	PGJ2					
20	Δ-12-PGJ2					
21	PGB2					
22	LTB4					
23	12-Keto-LTB4					
24	14,15-DHET	0.029	0.044	0.021		0.029
25	11,12-DHET	0.017	0.023	0.016		
26	8,9-DHET					
27	HXA3					
28	19-HETE					
29	20-HETE					
30	15-Deoxy-Δ-12,14-PGJ2					
31	5,6-DHET					
32	16-HETE					
33	15-HETE					
34	15-HPETE					
35	11-HETE	0.008	0.012	0.007	0.016	0.021
36	15-OxoETE					
37	12-HETE	0.087	0.077	0.107	0.081	0.135
38	8-HETE					
39	12-HPETE					
40	9-HETE					
41	12-OxoETE					
42	5-HETE					
43	5-HPETE					
44	14,15-EET					
45	5-OxoETE					
46	11,12-EET					
47	8,9-EET					
48	5,6-EET					

The amounts detected are shown in ng/mouse. Shaded columns indicate that the compounds were detected at negligible levels.
